# Local adaptation of both plant and pathogen: an arms‐race compromise in switchgrass rust

**DOI:** 10.1111/nph.70313

**Published:** 2025-06-22

**Authors:** Acer VanWallendael, Chathurika Wijewardana, Jason Bonnette, Lisa Vormwald, Felix B. Fritschi, Arvid Boe, Shelly Chambers, Robert B. Mitchell, Francis M. Rouquette, Yanqi Wu, Philip A. Fay, Julie D. Jastrow, John T. Lovell, Thomas E. Juenger, David B. Lowry

**Affiliations:** ^1^ Department of Horticulture North Carolina State University Raleigh NC 27607 USA; ^2^ Department of Plant Biology Michigan State University East Lansing MI 48824 USA; ^3^ Great Lakes Bioenergy Research Center Michigan State University East Lansing MI 48824 USA; ^4^ School of Integrative Plant Science Cornell University Ithaca NY 14850 USA; ^5^ Department of Integrative Biology University of Texas at Austin Austin TX 78712 USA; ^6^ Division of Plant Science and Technology University of Missouri Columbia MO 65201 USA; ^7^ Department of Agronomy, Horticulture & Plant Science South Dakota State University Brookings SD 57006 USA; ^8^ USDA‐NRCS, Kika de la Garza Plant Materials Center Kingsville TX 78572 USA; ^9^ USDA‐ARS Wheat, Sorghum, and Forage Research Unit University of Nebraska Lincoln NE 68583 USA; ^10^ Texas A&M AgriLife Research, Texas A&M AgriLife Research and Extension Center Overton TX 75684 USA; ^11^ Plant and Soil Sciences Department Oklahoma State University Stillwater OK 74078 USA; ^12^ USDA‐ARS Grassland Soil and Water Research Laboratory Temple TX 76502 USA; ^13^ Environmental Science Division Argonne National Laboratory Lemont IL 60439 USA; ^14^ Genome Sequencing Center HudsonAlpha Institute of Biotechnology Huntsville AL 35806 USA; ^15^ US Department of Energy Joint Genome Institute Berkeley CA 94720 USA; ^16^ Plant Resilience Institute Michigan State University East Lansing MI 48824 USA

**Keywords:** biofuel, BLUP, coevolution, fungal disease, GWAS, microbial ecology, rust, switchgrass

## Abstract

- In coevolving species, parasites locally adapt to host populations as hosts locally adapt to resist parasites. Parasites often outpace host local adaptation since they have rapid life cycles, but host diversity, the strength of selection, and external environmental influence can result in complex outcomes.
- To better understand local adaptation in host–parasite systems, we examined locally adapted switchgrass (*Panicum virgatum*), and its leaf rust pathogen (*Puccinia novopanici*) across a latitudinal range in North America. We grew switchgrass genotypes in 10 replicated multiyear common gardens, measuring rust severity from natural infection in a ‘host reciprocal transplant’ framework for testing local adaptation. We conducted genome‐wide association mapping to identify genetic loci associated with rust severity.
- Genetically differentiated rust populations were locally adapted to northern and southern switchgrass, despite host local adaptation to environmental conditions in the same regions. Rust resistance was polygenic, and distinct loci were associated with rust severity in the north and south. We narrowed a previously identified large‐effect quantitative trait locus for rust severity to a candidate YELLOW STRIPE‐LIKE gene and linked numerous other loci to defense‐related genes.
- Overall, our results suggest that both hosts and parasites can be simultaneously locally adapted, especially when parasites impose less selection than other environmental factors.

## Abstract 2

See also the Commentary on this article by Suffert & Marcel, **248**: 1108–1110.

## Introduction

Local adaptation, the process by which populations within a species adapt to narrow ranges of environmental conditions, is an important force maintaining intraspecific genetic and phenotypic diversity, especially in plant species (Kawecki & Ebert, 2004). Numerous cases of local adaptation have been described, and researchers have begun to uncover the genetic changes underlying fitness trade‐offs associated with local adaptation (Hall *et al*., 2010; Lowry & Willis, 2010; Anderson *et al*., 2011; Des Marais *et al*., 2013; Wadgymar *et al*., 2022). However, the majority of local adaptation research has focused on the influence of abiotic rather than biotic factors (Hargreaves *et al*., 2020). Theoretical treatment of biotic local adaptation has focused on host–parasite coevolution. In this field, researchers often use a tailored definition of local adaptation, wherein hosts or parasites can be considered locally adapted if they have higher fitness in the presence of their coevolved counterpart than in the presence of a foreign counterpart (Kaltz & Shykoff, 1998; Week & Bradburd, 2023). Host–parasite coevolution may result in arms‐races of local adaptation wherein selection is dominated by parasite virulence and host immunity, but local adaptation simultaneously occurs in response to the full ecology of other organisms and abiotic factors. Studying the genetic variation associated with local adaptation in both coevolving hosts and parasites across a geographic range can reveal evolutionary mechanisms that promote stable coexistence of locally adapted populations in dynamic biotic and abiotic conditions.

The balance between host and parasite coevolution is driven by differences in each species' population and quantitative genetic characteristics. In common host–parasite local adaptation models, each may evolve local adaptation to the other, yielding lower infection rates in coevolved populations if hosts are locally adapted, or higher infection rates in coevolved populations if parasites are locally adapted (Buckling & Rainey, 2002; Gandon & Michalakis, 2002; Greischar & Koskella, 2007). Parasites are expected to adapt more rapidly, given typically shorter generation times (Gandon & Michalakis, 2002). However, migration rates, clonality, and strength of selection play an essential role in determining whether the host or parasite locally adapts (Greischar & Koskella, 2007). Moderate migration in a parasite population can introduce new alleles and speed local adaptation (Gandon, 2002), although high migration rates decrease population barriers and lessen local differentiation (Kawecki & Ebert, 2004). Clonal reproduction in parasite populations reduces the effective population size and may therefore slow the pace of local adaptation (Gandon & Michalakis, 2002). Finally, the relative strength of selection plays an essential role. Host–parasite theory often focuses on parasites that prevent host reproduction (e.g. anther smuts in *Silene*), imposing high selection for host resistance that can result in parasite maladaptation (Kaltz *et al*., 1999). Given the many factors that can influence coevolution in host–parasite pairs, measuring local adaptation requires detailed knowledge of host and parasite ecology, as well as a robust experimental design.

The traditional test for local adaptation is a reciprocal transplant experiment (Kawecki & Ebert, 2004; Hereford, 2009; VanWallendael *et al*., 2022b; Wadgymar *et al*., 2022) in which individuals from each environment are transplanted to common gardens both in their local environment and foreign environments. Metapopulations, sets of populations connected by the movement of individuals, are considered locally adapted when the fitness of populations transplanted to a local environment is higher than fitness of foreign populations transplanted to that environment (local–foreign comparison; Kawecki & Ebert, 2004). When testing for local adaptation in parasites, researchers often replace the ‘environment’ with ‘host population’, and transplant parasites to different host populations in controlled settings (Kawecki & Ebert, 2004). An alternate method using a similar logic may be used to test for parasite local adaptation in natural settings, for which we propose the term ‘host reciprocal transplant’. A host reciprocal transplant involves moving host genotypes between regions with endemic parasite populations, avoiding common pitfalls of parasite local adaptation research and retaining essential biotic and abiotic context. Although this method has received less attention in local adaptation theory, it has been used in multiple experiments (Davelos *et al*., 1996; Laine, 2007; Busby *et al*., 2014; Cassetta *et al*., 2023). However, there are challenges to this method as well: Parasite populations must be both genetically differentiated and consistently present, and the interpretation must consider variable environmental effects on the both host and parasite (further details in the Materials and Methods section).

Local adaptation studies in parasites often use plant fungal pathogens, especially Puccineaceae rusts, owing to their narrow host range and high economic importance (Savary *et al*., 2019; Li *et al*., 2020). The population growth of rusts can depend on their both biotic and abiotic interactions. Rusts often have macrocyclic life cycles, with multiple hosts and five spore‐producing forms (Kolmer *et al*., 2009). The contribution of abiotic conditions to the success of rust infections varies somewhat between species, but generally freezing temperatures, low air turbulence, and dry conditions are less conducive to spread and infection (Helfer, 2014; Prank *et al*., 2019). Since all of these conditions are expected to shift with climate change, rust disease may pose greater challenges in the future (Dudney *et al*., 2021).

Wheat stem and leaf rusts have been widely researched, offering a model for host adaptation to multiple rust strains (Feuillet *et al*., 1995; McIntosh *et al*., 1995; Lillemo *et al*., 2008; Yu *et al*., 2014). Host plant resistance to fungal pathogens can take multiple forms ranging from resistance through a few immune‐related loci (Asnaghi *et al*., 2001; Salcedo *et al*., 2017) to polygenic resistance that includes structural or life‐history traits (Yu *et al*., 2019). Single‐gene resistance can be conferred by R‐genes such as *Sr35*, a wheat leucine‐rich repeat receptor (LRR) from *Triticum monococcum* that confers resistance to the Ug99 stem rust strain (Salcedo *et al*., 2017) by binding pathogen effectors to trigger the immunological hypersensitive response (HR; Förderer *et al*., 2022). Breeders distinguish seedling resistance from adult plant resistance. The latter describes resistance that may not be effective until later growth stages and does not involve HR, but typically provides resistance to more pathogen strains. Adult plant resistance is typically polygenic, consisting of multiple genes contributing smaller amounts to resistance (Aktar‐Uz‐Zaman *et al*., 2017). Despite rapid advances in understanding mechanisms of wheat rust resistance, knowledge of the degree to which these conclusions are transferable to other rust‐infected plants is lacking.

**Fig. 1:**
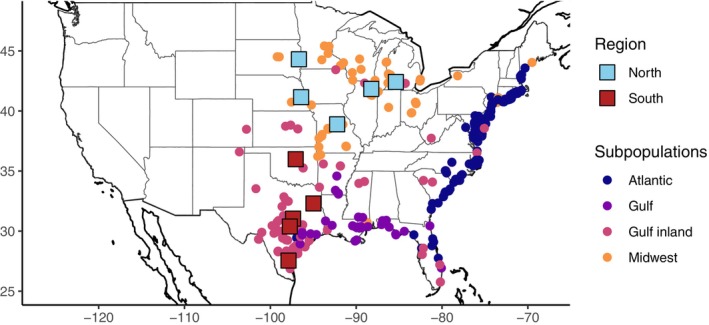
Collection locations (circles), and planting sites (squares) for the replicated switchgrass diversity panel. Coloring for circles indicates switchgrass population membership based on shared single‐nucleotide polymorphisms, and coloring for squares indicates the geographic region used for this manuscript. From north to south, the sites are Brookings, SD; Kellogg Biological Station, MI; Fermilab, IL; Lincoln, NE; Columbia, MO; Stillwater, OK; Overton, TX; Temple, TX; J.J. Pickle Research Campus, TX; and Kingsville, TX.

Switchgrass (*Panicum virgatum* L.) is a locally adapted perennial plant that is conducive to pathogen local adaptation studies, since it is consistently infected with several fungal pathogens, including a leaf rust (*Puccinia novopanici* Demers; Kenaley *et al*., 2019). Switchgrass genetic diversity is divided into three major genetic populations that mostly correspond with three ecotypes that are locally adapted to different ecoregions and habitats (Fig. 1). The Midwest population is adapted to the north‐central region of North America, the Atlantic population along the east coast, and the Gulf population in Texas and along the Gulf of Mexico (Kolmer & Liu, 2000). These populations differ greatly in phenotypic traits and stress resilience. Fitness trade‐offs that underlie local adaptation can be caused by genomic effects such as linkage, pleiotropy, or opposing conditionally neutral loci (Wadgymar *et al*., 2017), resulting in genotypes with variable stress tolerance across environments. The Gulf and Atlantic switchgrass populations are more susceptible to winter kill, due to lower freezing tolerance (Lovell *et al*., 2021; Willick & Lowry, 2022). Both typically have higher overall resistance to leaf fungi, although this can vary between years (VanWallendael *et al*., 2020). Previous research in a switchgrass quantitative trait locus (QTL) mapping common garden revealed that rust resistance patterns differ greatly between the northern and southern United States, although there was no difference in rust species composition across space (VanWallendael *et al*., 2020). Previously, we found large‐effect QTL for rust resistance on chromosomes 3N and 9N, but the outbred mapping population did not have sufficient resolution to narrow these to specific candidate genes.

In this study, we sought to understand regional differences in resistance and to more closely pinpoint genetic loci associated with rust infection severity using a genome‐wide association study (GWAS). Given large climatic differences as well as historical dominance of different ecotypes, we expected to find pathogen population genetic differentiation between northern and southern regions. Since previous research suggested that the upland ecotype is typically more susceptible overall to fungal pathogens (Uppalapati *et al*., 2013), we expected all pathogen populations would have the highest infection rates on the Midwest population rather than reciprocal local adaptation to different host populations. We also aimed to recapitulate previous results that rust severity was associated with two large‐effect loci in the north and several smaller effect loci in the south and to identify candidate genes involved in rust resistance. Finally, we aimed to identify potential pleiotropic loci by comparing GWAS results from rust severity with results for other vegetative and phenological traits.

## Materials and Methods

### Experimental design

This study used a replicated diversity panel experimental design described in Lovell *et al*. (2021). Briefly, switchgrass (*Panicum virgatum* L.) rhizomes were collected from numerous locations in the United States (Fig. 1). These rhizomes were clonally propagated at a facility in Austin, Texas, then transplanted to field sites in 2018, where we planted them in a honeycomb grid covered by weed‐cloth. Three sites, Austin, TX (PKLE), Columbia, MO (CLMB), and Kellogg Biological Station, MI (KBSM), were planted with all surviving 773 genotypes, and an additional seven sites were planted with a core subset of 630 tetraploid genotypes. The remaining octoploid and hexaploid genotypes were measured for rust (*Puccinia novopanici* Demers.) and other traits, but not used in the mapping population (Napier *et al*., 2022).

### Rust population genetics

In 2019, we collected rust‐infected leaves from nine of our sites (all except Overton, TX) to assess pathogen population structure. These samples were taken from adjacent (< 500 m) switchgrass experimental plots used for a previous mapping study (VanWallendael *et al*., 2020) that served as the presumptive source for rust propagules. At the Fermilab site (FRMI), we sampled from the main switchgrass diversity panel, since we did not have a previous experimental plot. We haphazardly collected 20 samples showing clear sori from each site. We dried the samples on silica gel and stored them at room temperature before processing. We scraped spores from sori, then extracted genomic DNA from each sample using a DNeasy Plant Mini Kit (Qiagen), following the manufacturer's protocol with modifications described in Kenaley *et al*. (2016). After DNA quality and quantity filtering, 88 samples remained with at least five samples from each site. We shipped samples to the Texas A&M Genomics and Bioinformatics Service for library preparation and sequencing on an Illumina NovaSeq 6000 (Illumina, San Diego, CA, USA). We sequenced the 99.9‐Mb genome to *c*. 50× coverage per sample using 150‐bp paired‐end reads.

Since *P. novopanici* does not grow in culture, we could not sequence single‐spore isolates. Samples from an individual plant were therefore assumed to contain a pool of multiple genotypes of the fungus. We aligned samples to the draft *P. novopanici* genome (Gill *et al*., 2019) using Burrows‐Wheeler Aligner (Bwa) and deduplicated alignments with Sambamba (Li & Durbin, 2010; Tarasov *et al*., 2015). Since the draft genome is highly fragmented (11 088 contigs; N50 = 13 091 bp), we performed a cleanup step by aligning contigs to a better‐quality *Puccinia triticina* reference genome (Wu *et al*., 2021). We used relaxed alignment parameters in Blast (−*e* value 1e‐5), and then discarded 1254 contigs that failed to map. Although a full revision of the *P. novopanici* genome is beyond the scope of this study, we used approximate contig positions in the *P. triticina* genome for visualizations. For single‐nucleotide polymorphisms (SNP)‐calling, we used BCFtools to generate an invariant‐sites Variant Call Format (VCF) file, and then filtered to biallelic sites with a minimum minor allele frequency (MAF) of 0.05 that were genotyped in all of our 88 samples using Plink2 (Li *et al*., 2009; Chang *et al*., 2015; Purcell & Chang, 2025). We used PoPoolation (Kofler *et al*., 2011) to assess diversity between regional pools. To group reads, we used SAMtools mpileup (Li *et al*., 2009). We then used *Variance‐sliding.pl* in PoPoolation to assess allelic diversity (π) using a sliding‐window approach. We used 1000‐bp windows with a 100‐bp step size. We filtered out reads with < 4× or > 70× coverage depth or an average Phred quality score < 20. We tested for differences in π between regions using the studentized bootstrap method suggested by Efron & Tibshirani (1994). We calculated the mean π values across windows for each contig to compare between regions, and then counted the number of contigs with π > 0.05 to assess the number of distinct outlier loci in each pool. We estimated *F*_ST_ between regions using the grenedalf package (Czech *et al*., 2023). Here, we used identical filtering steps but used individual contigs as windows, then computed the mean *F*_ST_ per window adjusted by the number of SNPs on each contig. We performed principal component analysis (PCA) on SNPs using singular value decomposition through the *big_SVD* function in the R package bigsnpr (Privé *et al*., 2018).

### Rust phenotyping and distribution

For rust severity, we followed a similar phenotyping protocol to that previously described in VanWallendael *et al*. (2020). Briefly, technicians checked experimental plants at each site daily for rust presence following spring green‐up. After the first instance of rust was detected, technicians scored plants four times over 8 wk, which was typically sufficient to capture the exponential growth phase of rust infection increase. We used a 0–10 scale, with each point corresponding to *c*. 10% of the total plant leaf area covered in rust sori. We calculated the area under the disease progression curve (AUDPC) for each plant for the 8‐wk period centered on the inflection point of rust increase at each site. The AUDPC is a commonly used metric that accounts for differences in rates of disease progression to produce a single measurement that can be thought of as ‘disease severity’. Additional phenotypic measurements were taken for switchgrass plants as described in Lovell *et al*. (2021), including height, tiller number, flowering time, and biomass. We visualized phenotypic clustering in switchgrass populations using rust severity and other phenotypes via PCA. We examined three focal sites (KBSM, CLMB, and PKLE) that had the greatest number of shared host genotypes (*n* = 1070). We used the mean value of each trait for each genotype across sites and years in the PCA. We removed 94 genotypes with missing data, and then computed the PCA using the centered and scaled values in the *prcomp* function in R.

We assessed rust distribution across host genotype, space, and time using linear mixed models (LMMs) in the R package sommer (Covarrubias‐Pazaran, 2016). Since a model including all sites failed to converge, we split sites into northern (BRKG, KBSM, FRMI, and LINC) and southern (CLMB, OVTN, TMPL, PKLE, and KING) regions, reflecting the approximate distribution of the Gulf and Midwest populations (Fig. 1). We used the *mmer* function to solve the following model with a compound symmetry variance–covariance matrix separately for each region, with AUDPC as the response variable and random effects for site, year, genotype, and the genotype‐by‐environment (site–year combinations) interaction. We evaluated the importance of model components by sequentially dropping each term and testing for fit differences using a likelihood ratio test via the *anova* function in sommer.

AUDPC~1+1∣site+1∣year+1∣genotype+1∣genotype:environment

#### The host reciprocal transplant for rust local adaptation

**Fig. 2:**
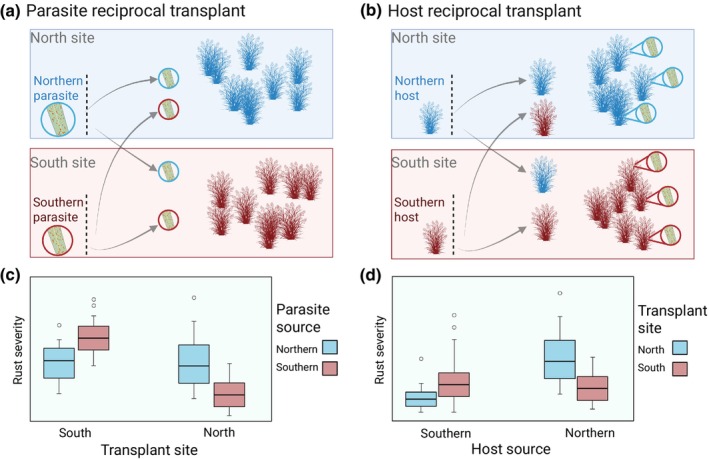
Comparing two methods for testing parasite local adaptation, a parasite reciprocal transplant (AC) and a host reciprocal transplant (BD; this study). In this example scenario, two host populations (switchgrass plant icons) and two parasite populations (circular leaf rust icons) are differentiated between two sites, North and South. (a) Traditional parasite reciprocal transplant would introduce northern and southern rust into switchgrass populations at each site. (b) A host reciprocal transplant would introduce northern and southern switchgrass in a common garden to endemic rust populations at each site. (c) Proof of parasite local adaptation via the local–foreign comparison would require greater fitness for southern rust over northern rust on southern switchgrass, and greater fitness for northern rust over southern rust on northern switchgrass. This is indicated by greater rust severity from southern rust (red boxes) in the south site, but greater rust severity from northern rust (blue boxes) in the north site. (d) Proof of parasite local adaptation would require the same fitness advantage as in a parasite transplant: northern rust over southern rust on northern switchgrass, and greater fitness for southern rust over northern rust on southern switchgrass. However, this test would be within host populations rather than within sites, as indicated by swapping the transplant site to the legend from the *x*‐axis. Boxplots shown in (c, d) are shown for demonstration and do not include real data; they are drawn to depict outliers as points, 1.5 × the interquartile range as whiskers, the 25^th^ and 75^th^ percentiles as upper and lower box limits, and the median as the center line. This figure was created in BioRender (BioRender.com/8nb3jl8).

We used a modified version of the classic local adaptation test to assess parasite local adaptation, which we have termed a host reciprocal transplant (Fig. 2). While the host transplant is similar in concept to traditional reciprocal transplants, it differs in what is considered the transplant ‘habitat’ when defining what is local and foreign. When assessing host–parasite local adaptation, each coevolutionary partner can be considered the essential ‘habitat’ for the other (Kawecki & Ebert, 2004). The host reciprocal transplant can therefore be considered a reciprocal transplant in which the biotic environment (host) is transplanted, rather than the adapting population. Fewer regulatory and ethical issues surround transplant of hosts than their parasites, and unculturable parasites may be particularly challenging to transplant. A host reciprocal transplant overcomes some of the challenges of translating controlled common garden experiments to the field. Growth chambers and other mesocosms typically remove much of the environmental context of the host–parasite interaction, and both susceptibility and infectivity can be context‐dependent. Similarly, controlled experiments select for, and are biased by genotypes that are simply better‐adapted to laboratory or glasshouse conditions (Kawecki & Ebert, 2004). However, environmental context is quite different between parasite and host reciprocal transplants. Is local context more important for the parasite infectivity or the host susceptibility? If we make a general assumption that both host and parasite are well‐adapted to their native biotic and abiotic environment, transplanting each outside their adapted range should tend to weaken populations, making hosts more susceptible and parasites less infective. Therefore, in a host transplant experiment, we would tend to underestimate the strength of parasite local adaptation, since foreign parasite populations infect hosts that have lowered susceptibility. By contrast, parasite reciprocal transplants may overestimate parasite local adaptation, since foreign parasites may have reduced infectivity in novel environments. A reversed pattern may be possible in systems in which organisms are maladapted to their local environment, or if interactions with other organisms such as hyperparasites make foreign transplant advantageous.

To specifically test the hypothesis that rust is locally adapted to switchgrass, we assessed whether rust susceptibility was higher in switchgrass genotypes challenged with coevolved pathogens. Since rust pathogen pressure can be variable across years and different technicians rated rust severity across sites using a relatively subjective metric, finding the true genetic basis of rust resistance required minimizing other sources of variation. When combining data across environments, genetic mapping studies often calculate best linear unbiased predictions (BLUPs) for each genotype, and then use these as a phenotype for GWAS (Wallace *et al*., 2016; Kumar *et al*., 2018; Cui *et al*., 2021). We used the above‐described LMM to calculate rust severity BLUPs for northern and southern regions. BLUPs correlate well with the mean of centered and scaled AUDPC genotype scores for both northern and southern sites (*r* = 0.87 and *r* = 0.94, respectively). Using a kinship matrix instead of genotype identity in the model produced highly similar BLUPs (*r* = 0.99 both regions), so we only used genotype identity. Some recommendations suggest using best linear unbiased estimators (BLUEs) instead of BLUPs in two‐stage GWAS models (Holland & Piepho, 2024). We estimated BLUEs by including genotype as a fixed rather than random effect but found a very close correlation between BLUPs and BLUEs (*r* = 0.99) and no difference in top SNP outliers, so used BLUPs for the remainder of the study. Since BLUPs were not normally distributed (Shapiro–Wilk *P* < 0.0001), we used a nonparametric Dunn's test to assess differences in means between groups when testing local adaptation, with a Bonferroni correction for multiple testing.

### GWAS: the genetic basis of switchgrass rust resistance

In our analyses, we used a set of switchgrass SNPs first generated by Lovell *et al*. (2021). This set comprised 10.8 million SNPs after quality filters and a MAF cutoff of 0.05. We performed a PCA using the *big_SVD* command in the R package bigstatsr (Privé *et al*., 2018). We computed GWAS using BLUPs from northern and southern sites through the *pvdiv_gwas* function in the switchgrassgwas R package (Lovell *et al*., 2021). This function runs linear regression on file‐backed big matrices, with 10 principal components as covariates to correct for population structure. As an additional correction for population structure, we repeated GWAS runs in each switchgrass subpopulation by subsetting the data into three population groups using PC1 from the PCA.

We assessed the top genes linked to GWAS outliers using JGI JBrowse (Skinner *et al*., 2009), NCBI Blast, and NCBI Genbank. We considered genes < 10 kb from a lead SNP as linked to the SNP, since this is the typical distance of linkage decay in the switchgrass genome (Supporting Information Fig. S1). We assessed which gene functions were overrepresented in our dataset using a bootstrapping method focused on the 20 gene functional annotations with the greatest difference in frequency between the sample and total dataset. We randomly permuted genes from the total genome‐wide list for 100 000 iterations, and then assessed whether each function was more common than the permuted set.

To assess *in situ* expression of candidate genes, we used data from a previously described RNA‐sequencing experiment (VanWallendael *et al*., 2022a). Briefly, RNA was assayed from leaf tissue that was (1) excised at the ligule, (2) 2 cm of the proximal portion of the excised leaf were separated from the midrib, (3) placed in a 2‐ml Eppendorf tube loaded with three stainless steel beads, (4) immediately frozen in liquid nitrogen, and (5) transported on dry ice to the laboratory. Tissue was homogenized with a GenoGrinder 2000. RNA was extracted with the standard Trizol protocol and treated with DNase I to remove DNA contamination, then sequenced on an Illumina HiSeq 2500. We examined four genotypes: two that are typically rust‐susceptible (DAC6 and VS16) from the upland ecotype and two that are more resistant (AP13 and WBC3) from the lowland ecotype. We assessed differential expression (DE) using the DESeq2 package in R (Love *et al*., 2014). We filtered out genes that had counts lower than 10 for three or more samples, according to best practices (Love *et al*., 2014). To test for enrichment of GWAS hits linked to DE genes, we followed the permutation method of Lasky *et al*. (2014), in which linkage between the top 5% of SNPs and DE genes are compared with the distribution of 10 000 random draws of the same number of genes from all possible switchgrass genes.

Finally, we assessed the genetic correlation of rust to other switchgrass phenotypes using LMMs in R. We fit three models for biomass, flowering time, and tiller count that included environment (site–year combination) and a kinship matrix with the formula:

$$\left(\text{AUDPC},\text{trait2}\right)\sim 1+1|\text{site}\_\text{year}+1|\text{kinship}$$

Biomass and tiller count were taken at the end of the season and represent the dry mass of aboveground leaf tissue and the number of tillers (stems), respectively (Lovell *et al*., 2021). Flowering time was assessed as the day of year when half the crown had flowered. In order to identify potential pleiotropic loci, we repeated GWAS mapping with each of the three traits, and examined overlap with rust GWAS.

## Results

### Rust populations differ between Northern and Southern US

**Fig. 3:**
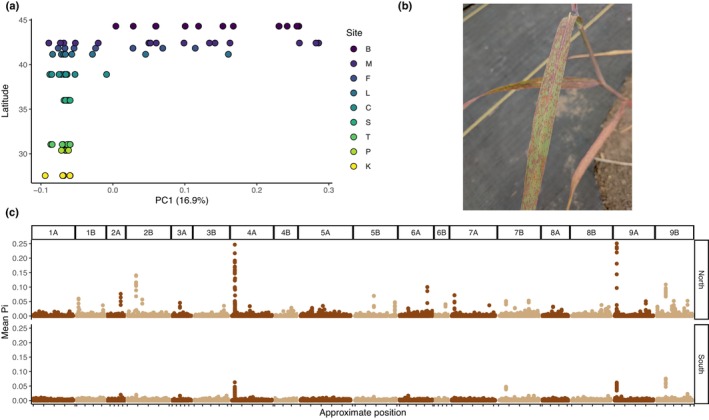
*Puccinia novopanici* leaf rust population genetics across nine sites. (a) The *y*‐axis indicates the latitude of the collection site, and the *x*‐axis is the first principal component of a principal component analysis of 2.4 million single‐nucleotide polymorphisms, indicating genetic similarity. Site codes correspond to: B – Brookings, SD; C – Columbia, MO; F – Fermilab, IL; K – Kingsville, TX; L – Lincoln, NE; M – Kellogg Biological Station, MI; P – J.J. Pickle Research Campus, TX; S – Stillwater, OK; T – Temple, TX. (b) *Puccinia novopanici* sori under field conditions in Kingsville, TX. Photograph by Acer VanWallendael. (c) Mean nucleotide diversity (Pi) across 10‐kb windows in the *P. novopanici* genome. Positions are shown by mapping location in the *Puccinia triticina* genome.

To understand the genetic structure of rust populations, we used whole‐genome resequencing on samples from rust populations at nine sites. Across 88 samples, this resulted in a set of 2.4 million SNPs with a minimum MAF of 0.05 that were called in all samples. Genetic PCA revealed that samples from northern and southern sites were mostly distinct (Fig. S2A) and that PC1 was correlated with latitude of collection (Fig. 3a).

To uncover differences in genetic diversity between rust populations, we scanned the genome for regions of increased diversity using pooled sequencing analyses. In this analysis, individual and site differences are combined to highlight large‐scale differentiation across the genome. Since rust dispersal within a season occurs mostly through asexual urediniospores, we expected to find mostly low‐diversity clones. However, high‐diversity windows may indicate the emergence of clones with novel mutations in fitness‐related genes. We examined northern and southern regional pools (Fig. 3c) for loci with increased diversity that may indicate selection. One large outlier locus with π > 0.75 on chromosome 9B was linked to the Internal Transcribed Spacer (ITS) region. Since ITS regions often exist as large tandem repeats, the very high diversity at this locus is likely spurious, so we excluded this region from further analysis. Overall genome‐wide π was 4.7% higher in the North (northern π = 7.97 × 10^−4^, southern π = 7.61 × 10^−4^, bootstrap *P* = 0.0006). Additionally, northern populations contained 15 unique outlier loci (π > 0.05; Fig. 3c), whereas the southern populations contained just three outliers. Genome‐wide average *F*_ST_ between regions was moderately high at 0.141 but did not show clear regions of differentiation, possibly owing to poor genome quality in this species (Fig. S2B). While gene annotation exists for some *Puccinia* species, functional annotation is limited to a few well‐conserved genes, so we were not able to confidently link outliers to known rust genes.

### Rust is locally adapted in northern and southern switchgrass populations

We used the diverse source material planted at multiple locations to measure both variation in the genotypic component (source genotype), and environmental component (year and planting site) of the expression of susceptibility to rust. To compare the relative amounts of variation attributable to genotype, site, year, and genotype × environment interaction (G×E), we fit an LMM for AUDPC across sites and years in northern and southern regions. For both regions, the highest variance components were genotype (northern = 0.244, southern = 0.297; Table 1) and G×E (northern = 0.264, southern = 0.109). Overall, the model explains 63.2% of the variation in AUDPC in northern sites (AIC = 3788), and 55.5% of variation in southern sites (Akaike information criterion (AIC) = 2550). Dropping each of the terms from models resulted in significantly poorer model fit (Likelihood Ratio Test; Table 1).

**Table 1:** Variance proportions for linear mixed models in northern and southern regions for rust severity on switchgrass.

Northern				
	Prop. variance	*Z* ratio	LRT ChiSq	LRT *P* value
Site	0.0772	1.21	432.8	< 0.001
Year	0.0472	0.99	220.6	< 0.001
Genotype	0.2439	12.75	621.4	< 0.001
G×E	0.2635	6.67	16.2	< 0.001
Residual	0.3682	9.62		

LogLik	AIC	BIC		
−1893	3788	3794		

Southern				
	Prop. variance	*Z* ratio	LRT ChiSq	LRT *P* value
Site	0.107	0.99	327.9	< 0.001
Year	0.042	0.99	172.2	< 0.001
Genotype	0.297	12.93	891.0	< 0.001
G×E	0.109	2.49	5.7	0.017
Residual	0.445	10.17		

LogLik	AIC	BIC		
−1274	2550	2572		

LRT columns show the results of a likelihood ratio test that compares a full model and a model without each term. The G×E term represents a genotype‐by‐environment interaction, with each site‐year combination as a separate environment. LogLik, AIC, and BIC refer to the log‐likelihood, Akaike information criterion, and Bayesian information criterion for each model, respectively.

**Fig. 4:**
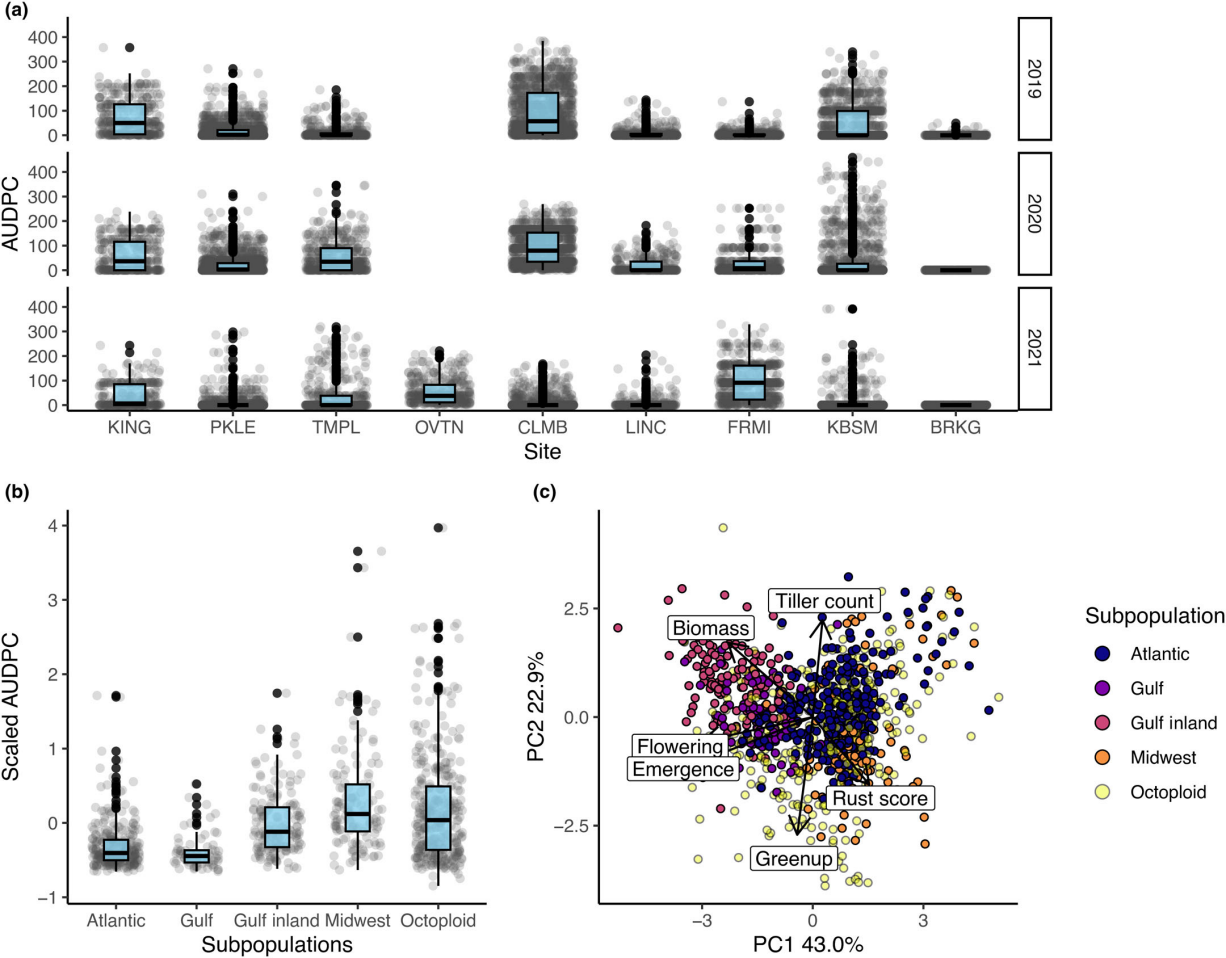
Rust score variation across switchgrass populations, space, and time. (a) Site and year variation in rust infection on switchgrass. Points shown indicate the area under the disease progression score across 8 wk for each plant in each year. Sites are shown on the *x*‐axis; from north to south, the sites are BRKG (Brookings, SD), KBSM (Kellogg Biological Station, MI), FRMI (Fermilab, IL), LINC (Lincoln, NE), CLMB (Columbia, MO), OVTN (Overton, TX), TMPL (Temple, TX), PKLE (J.J. Pickle Research Campus, TX), and KING (Kingsville, TX). 2019–2020 data could not be collected at OVTN (Overton, TX) and rust was not found in BRKG in 2020–2021. (b) Genetic subpopulations vary in rust severity. Points indicate mean scaled rust severity (area under the disease progress curve (AUDPC)) across sites and years. The Gulf genetic population has a subdivision between genotypes originating inland and those by the coast. Boxplots show outliers as points, 1.5 × the interquartile range as whiskers, the 25^th^ and 75^th^ percentiles as upper and lower box limits, and the median as the center line. (c) Phenotypic principal component analysis biplot for major traits across all sites and years. Points are colored by subpopulation.

Different technicians rated rust severity at each site, so the variance explained by site may be partially attributable to subjective rating differences (Fig. 4a). Some sites had consistently high rust ratings, such as CLMB (Columbia, MO), and others varied between years such as in BRKG (Brookings, SD), which had no rust in 2020 or 2021. To reduce this bias when summarizing across genotypes, we centered and scaled rust severity scores before comparing populations (Fig. 4b). Rust varied greatly between genetic subpopulations (Fig. 4b) and covaried with several other traits, particularly time of plant green‐up and biomass (Fig. 4c). The Gulf switchgrass population was split between genotypes originating from the Gulf coast and those from more inland, mostly in Texas, so these subgroups are separated in most analyses. Octoploid genotypes, which can be found across the switchgrass range, had the greatest overall variation. In a trait PCA (Fig. 4c), biomass was negatively correlated with rust scores, with higher biomass present in the central Texas population, and higher rust scores in the Midwest. Green‐up date was more closely correlated with rust score, and traded off with tiller count.

**Fig. 5:**
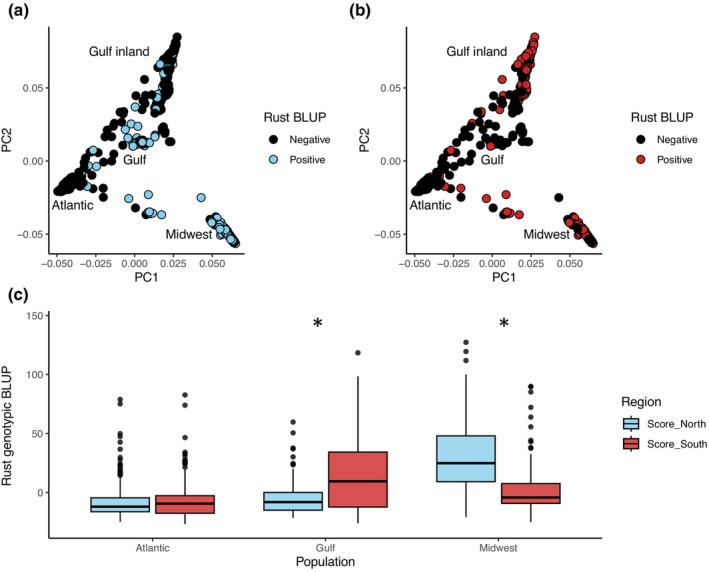
Evidence for rust local adaptation to switchgrass across two regions. Switchgrass genomic principal component analysis showing susceptibility to endemic rust in northern (a) and southern (b) sites. Rust severity best linear unbiased predictor (BLUP) was measured in northern and southern sites. Negative scores indicating genotype resistance are shown as black, positive scores indicating genotype susceptibility are colored. (c) Rust BLUPs by host population and region. Blue boxes indicate samples planted in northern common gardens, red boxes indicate samples planted in the southern gardens. Asterisks indicate regional differences; Dunn's test *P* < 0.0001 for both. Boxplots show outliers as points, 1.5 × the interquartile range as whiskers, the 25^th^ and 75^th^ percentiles as upper and lower box limits, and the median as the center line.

To test the hypothesis that rust pathogens are locally adapted to switchgrass genotypes, we examined differences in rust severity for genotypes across locations using BLUPs from LMMs. BLUPs estimate switchgrass genotypic contribution to rust severity, with higher values indicating genotypes with greater susceptibility to rust across sites and years. Individuals from the Midwest population were commonly susceptible to rust present in northern sites (positive BLUP; Fig. 5a), whereas those in the Gulf inland subpopulation were more susceptible when infected with rust in southern sites (Fig. 5b). Northern and Southern populations of rust therefore conformed to expectations of local adaptation, resulting in higher rust severity on Midwest and Gulf switchgrass populations, respectively (Fig. 5c; Dunn's test: Bonferroni‐adjusted *P* < 0.0001 for both populations). By contrast, rust severity was not differentiated in genotypes from the Atlantic population, which did not coevolve with pathogens at any of our transplant sites (Fig. 5c; Dunn's test: Bonferroni‐adjusted *P* > 0.999).

### The polygenic genetic basis for resistance differs across regions

**Fig. 6:**
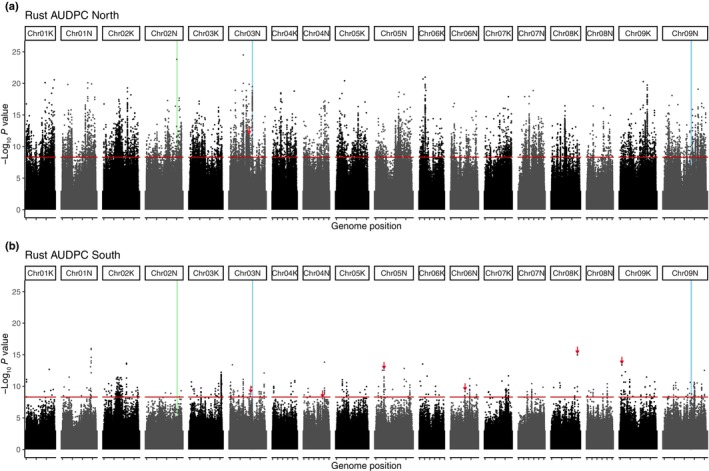
Genome‐wide association study (GWAS) of disease severity best linear unbiased predictors in northern (a) and southern (b) regions. Vertical lines indicate the positions of candidate loci found in previous experiments (VanWallendael *et al*., 2020, 2022a). The green line on chromosome 2N indicates a microbiome structure – associated GWAS outlier, and blue lines indicate rust resistance quantitative trait loci *Prr1* (Chr03N) and *Prr2* (Chr09N). Red horizontal lines indicate a Bonferroni cutoff. Red arrows indicate points that were also identified as outliers in population‐specific GWAs.

We conducted switchgrass GWAS separately for the northern and southern regions, using rust severity BLUPs as predicted traits in a linear GWAS model. We found numerous loci associated with rust severity, indicating a largely polygenic response (Fig. 6). In both northern and southern regions, *P*‐values showed signs of inflation, despite a conservative correction with 10 PCs (North lambda = 1.042, South lambda = 1.092; Fig. S3). When we ran GWAS within subpopulations, we found the reverse; smaller sample sizes yielded fewer significant SNPs. For all significant SNPs shared between regions, there was only a weak positive correlation between −log_10_ *P*‐values (*r* = 0.354; *P* < 0.0001), indicating few shared mechanisms. Indeed, the correlation disappears when examining only the top SNPs in each region.

We examined genes linked to the top 100 lead SNPs in the northern and southern sites, as well as genes linked to SNPs that were outliers in both the subpopulation‐specific and overall analyses. There were no overlaps between top SNPs or the genes linked to them between regions. We used enrichment analysis to determine which annotations were overrepresented. While most of the enriched annotations did not have a clear link to disease resistance, there were some exceptions. In the north, four gene annotations were overrepresented in our top SNPs (Fig. S4). Terpenoid cyclases, which allow formation of specialized metabolite defenses such as antifungal leaf saponins, were common (*P* < 0.001). In the south, the most notable of the five overrepresented annotations were EF‐TU receptors, LRRs that typically recognize bacterial pathogens (Schoonbeek *et al*., 2015). Outliers on chromosomes 2N (North), 3K (South), 4K (South), and 6K (South) were closely linked to LRRs. A multidrug and toxin extrusion transporter is linked to a lead SNP on chromosome 1K in the south. These genes can function in pathogen responses by moving hormones such as salicylic acid in response to infection (Serrano *et al*., 2013). Although fewer outlier SNPs overall were identified in the South, more loci matched between subpopulation‐specific and overall GWAS analyses. However, none of the nearby genes had clear functions related to pathogen resistance.

Previous research identified several loci associated with switchgrass–fungal interactions (Milano *et al*., 2016; VanWallendael *et al*., 2020, 2022a). The strongest candidate gene we identified within the chromosome 3N *Prr1* QTL region was Pavir.3NG168388 (Fig. S5). This gene is an oligopeptide transporter similar to YELLOW STRIPE‐LIKE, which not only functions in metal ion distribution in plants (Sheng *et al*., 2021) but also confers pathogen susceptibility when mutated (Chen *et al*., 2014). A nearby Acyl CoA acyltransferase (Pavir.3NG162000) was also within the 3N *Prr1* QTL region and was identified as the only subpopulation‐specific outlier for the Northern region. Acyltransferases have diverse roles in stress response and other cellular functions (Lou *et al*., 2016), so the presence of this gene does not suggest its specific role in immunity. The *Prr2* QTL region on 9N contained relatively weaker GWAS hits, but one of these was linked to the highly conserved CCR4‐NOT complex (Pavir.9NG474500), which acts as a multifunctional gene expression regulator (Collart & Panasenko, 2017). A large outlier on chromosome 2N is near a previously discovered cluster of cysteine‐rich receptor‐like kinases associated with variation in the leaf microbiome (VanWallendael *et al*., 2022a). This outlier is linked more closely, however, to a cluster of four genes with predicted functions in drug and disease resistance (Fig. S5).

**Fig. 7:**
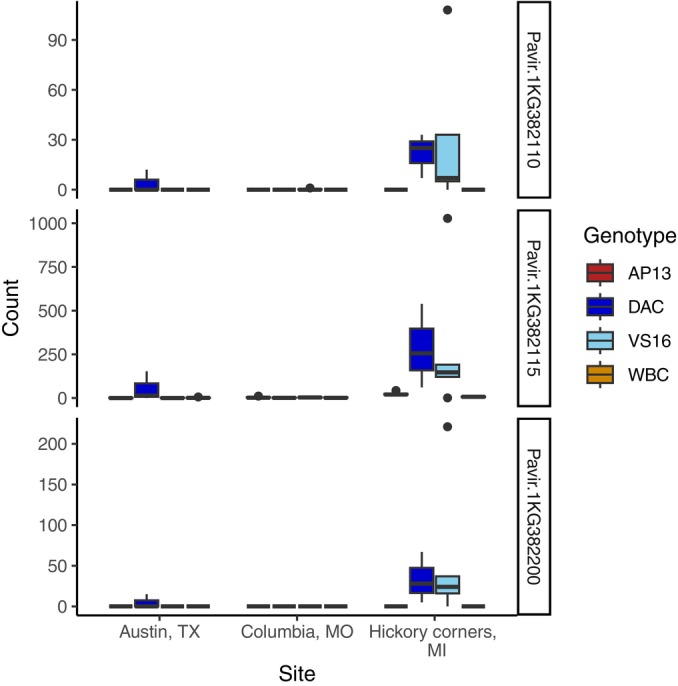
Three terpenoid cyclase genes were expressed almost exclusively in switchgrass leaf tissue from northern upland switchgrass varieties grown in northern sites in 2016. These three genes are linked to large genome‐wide association study outliers on chromosome 1K. AP13 and WBC are southern genotypes in the Gulf population; DAC and VS16 are northern genotypes in the Midwest population. Boxplots show outliers as points, 1.5 × the interquartile range as whiskers, the 25^th^ and 75^th^ percentiles as upper and lower box limits, and the median as the center line.

To test the hypothesis that these candidate genes underlie divergent switchgrass responses to northern and southern rust genotypes, we analyzed differences in their transcription in switchgrass leaves at three of our sites, KBSM, CLMB, and PKLE in four genotypes, two from the northern upland ecotype, and two from the southern lowland ecotype. Overall, 12 151 of 52 849 genes were DE between ecotypes, and these were enriched for rust‐associated GWAS SNPs (*P* = 0.0116 North, *P* = 0.0120 South). Of the 107 genes closely linked to top GWAS hits in the North, 82 were DE between resistant and susceptible varieties (top 20 in Table S1). A gene with unknown function (Pavir.7NG105945) was most clearly differentiated, followed by a gene on 1N in the RING/U‐box superfamily (Pavir.1NG505000). The oligopeptide transporter underlying the *Prr1* QTL had very low expression in the leaf for the four genotypes in this study, but the gene cluster on chromosome 2N was DE. Three terpenoid cyclases with putative roles in antifungal metabolite production on chromosome 1K were more strongly expressed in northern upland genotypes. Strikingly, they showed much higher expression in the northernmost site, suggesting that these three genes may be upregulated in response to either infection by particular strains of fungi or other environment‐specific stimuli (Fig. 7). For southern outliers, 96 of 130 genes linked to top GWAS hits were DE. The strongest differentiation was seen in a small ribonucleoprotein F gene on chromosome 5N (Pavir.5NG167600; Table S1), but an LRR detected on chromosome 3K (Pavir.3KG551700) was clearly differentiated as well.

### Outlier loci are rust‐specific

To determine the extent to which variation in rust severity is linked to other switchgrass phenotypes, we assessed genetic correlations with biomass, flowering time, and tiller count using LMMs. As was suggested in a trait PCA (Fig. 4c), all three traits had negative genetic correlations with rust severity. Flowering time had the strongest correlation (−0.410) followed by tiller count (−0.342) and biomass (−0.254). To predict whether genes associated with rust have pleiotropic effects on other switchgrass traits, we additionally performed GWAS on four additional traits: biomass, tiller number, flowering date, and green‐up date. Of these, only biomass yielded outlier loci above the Bonferroni cutoff, but none were shared with rust. The recommended method for evaluating concordance between GWAS tests is a circular permutation, which permutes *P*‐values across chromosomes while keeping loci order constant (Cabrera *et al*., 2012). However, this test requires at least one shared significant *P*‐value to be meaningful, so we were not able to test this concordance directly.

## Discussion

In common theoretical models of host–parasite coevolution, either hosts or parasites may evolve local adaptation to each other. While theory predicts that parasites with short generation times will locally adapt across the geographic range of the host more quickly than the host can evolve defenses (Gandon & Michalakis, 2002), the pattern has only rarely been observed in natural populations. In ecosystems, host–parasite coevolution occurs in the context of a multitude of additional biotic and abiotic effects that can influence the results of a coevolutionary arms‐race. Our results indicate a less‐explored intermediate result that both host and parasite evolve local adaptation: the host to regional environmental conditions and the parasite to specific host populations. Previous research supports local adaptation of our host, switchgrass, to northern and southern regions of North America (McMillan, 1965; Casler *et al*., 2004, 2007). However, we found that the rust pathogen is also locally adapted to host populations derived from the same regions, indicating that host and parasite local adaptation are not mutually exclusive in this system. In an earlier QTL study, we found that two large‐effect loci were primarily responsible for variation in rust susceptibility in northern field sites (VanWallendael *et al*., 2020). Here, using GWAS, we instead found a highly polygenic architecture underlying rust susceptibility in both northern and southern regions. Below, we address several explanations for these two main findings.

In the northern and southern United States, we found that rust is locally adapted to switchgrass, which is itself locally adapted to distinct ecoregions in North America. This outcome may be attributed to a low inverse correlation between host and parasite fitness (Salvaudon *et al*., 2005, 2007), in this case relatively low damage imposed on switchgrass from rust. In a similar study that also found local adaptation in both a rust and grass plant, *Holcus* plants experienced the greatest rust infection later in their life cycle, allowing parasites to gain fitness without imposing a strong cost on their host (Crémieux *et al*., 2008). Consistent with that explanation, rust infections often reach high loads in switchgrass populations, but rarely cause mortality or full loss of fitness (VanWallendael *et al*., 2020). By contrast, freezing temperatures in the northern Midwest region of the United States are thought to act as a major force driving local adaptation in switchgrass (Lovell *et al*., 2021). Proportionally greater impacts from freezing on switchgrass fitness than rust may prevent switchgrass from being locally maladapted overall in regions where rust is also locally adapted.

The pattern of local adaptation in both species has implications for understanding the genetic structure of switchgrass' response to rust. While disease resistance loci often show large effect sizes from just one or a few candidate genes, as is the case for wheat resistance to rust, the nature of pathogen selective pressure can influence resistance genetic architecture (Lemoine *et al*., 2012). Since pathogen selection pressure is low, a rust resistance locus linked to a freezing tolerance locus (for instance) would be ineffectively selected, remaining at intermediate allele frequencies in a classic Hill–Robertson effect (Charlesworth, 2012; Lotterhos *et al*., 2018). In an association study, this phenomenon tends to result in lowered association scores for linked loci, potentially obscuring causal variants. The highly polygenic association between switchgrass and rust in northern and southern populations may also be partially an artifact of high population structure in switchgrass. While QTL mapping eliminates population structure through controlled crossing, GWAS must contend with loci that are correlated with population structure when populations show mean differences in the trait of interest (Vilhjálmsson & Nordborg, 2013). We minimized this effect in our study by using a strong correction for structure in the inclusion of 10 PCs in our model, comparing results to the inclusion of a kinship matrix, and recalculating GWAS in each subpopulation. If population structure is not adequately controlled, GWAS for multiple traits with mean differences across populations will show spurious loci that are correlated across GWASs (Veller & Coop, 2024). The fact that other switchgrass phenotypic traits had a distinct genetic architecture from rust is an indication that structure did not unduly bias our results.

Alternatively, a highly polygenic response may be indicative of a particular type of resistance (or susceptibility). While relatively few studies have mapped the genetic basis of susceptibility to a locally adapted plant parasite, polygenic resistance has been found in previous cases (Bellis *et al*., 2020). Plant quantitative resistance often comes from the combined action of numerous genes, especially in cases of structural resistance (González *et al*., 2012). More generalized pathogen resistance through protective specialized metabolites could be selectively favored when pathogen populations evolve rapidly, since strain‐specific R‐genes would be quickly defeated (Hulse *et al*., 2023). Our results suggest a polygenic basis of adaptation, and we found several genes related to the production of specialized metabolites. This included terpenoid cyclase genes related to the mevalonate or MEP synthesis pathway, which were overrepresented in our results. Recent research on switchgrass has shown that steroidal saponins produced by this pathway are essential to the species' resistance to fungal pathogens (Li *et al*., 2023). Thus, the many genes associated with rust we uncovered through GWAS may reflect different aspects of the production, distribution, and storage of these chemicals.

Mechanisms underlying host–parasite dynamics in natural populations are more complex than what is seen in simulations or in controlled conditions. Variable selection from both the biotic and abiotic environment results in patterns of local adaptation that may not be predictable from just 1 yr of data (Kawecki & Ebert, 2004; Dittmar & Schemske, 2023). Our use of a ‘host reciprocal transplant’ allowed us to test for local adaptation among parasite populations using an experimental design that lacks many of the drawbacks of other study designs. By reciprocally transplanting hosts rather than parasites, we incorporated natural variation in pathogen strains, along with the native environment of pathogens. In the correct conditions, future studies can benefit from similar designs for examining host–parasite coevolution.

Some challenges with experimental design may have influenced our results. In particular, evaluating infection status consistently across multiple years and thousands of kilometers may have resulted in greater noise in trait data than would be ideal. Advances in aerial imaging have been used in other studies to more clearly quantify infection data, and may be useful in similar future experiments (Shakoor *et al*., 2017). Furthermore, we were only able to capture 1 yr of variation in rust population genetics. It is likely that the border between northern and southern rust populations varies from year to year, so we might expect local adaptation to be less clear in transplant sites from intermediate latitudes. Assessments of the overall leaf microbial community in switchgrass common gardens have indicated that the latitudinal cline in rust diversity is mirrored by a distinction in the overall leaf microbiome between northern and southern sites, although whether this is a correlate, cause or effect of rust strain variation is unclear (VanWallendael *et al*., 2022a). Another attempt to quantify switchgrass rust strain diversity focused on the southeast region but does indicate a distinct haplotype present in their single Midwest population (Bahri *et al*., 2025).

This study reinforces the importance of studying host–parasite interactions in natural systems, to uncover the extensive variation and surprising results that can be revealed with long‐term experiments that include environmental context (Cocciardi *et al*., 2024). Understanding the host and environmental factors that drive parasite ranges will be critical in predicting parasite shifts under climate change. The host reciprocal transplant is an underutilized tool for this critical study. Here, it has revealed that one common portrayal of a zero‐sum outcome from a host–parasite arms race is not an inevitable outcome of coevolution, but that the relatively weak selection imposed by the rust pathogen allows local adaptation in the same geographic regions. In such a system, field experimentation can provide clear insights into the delicate balance of natural selection in large coevolving metapopulations, and illustrate how both hosts and parasites respond to changing selection.

## Competing interests

None declared.

## Author contributions

AV, CW, JB, TEJ and DBL planned and designed the experiment. JB, LV, FBF, AB, SC, RBM, FMR, YW, PAF, JDJ, TEJ and DBL managed field plantings and maintenance. AV, CW, JB, JTL and LV collected data. AV, CW and JTL analyzed data. AV, TEJ and DBL wrote the manuscript.

## Disclaimer

The New Phytologist Foundation remains neutral with regard to jurisdictional claims in maps and in any institutional affiliations.

## Supporting information


**Fig. S1** Linkage decay in each switchgrass subpopulation.
**Fig. S2** Principal component analysis and genome‐wide *F*_ST_ calculations for rust samples.
**Fig. S3** Quantile‐quantile plots for northern and southern sites genome‐wide association study.
**Fig. S4** Top overrepresented gene functions linked to outlier loci in the North and South regions.
**Fig. S5** Outlier‐linked regions on Chromosomes 3N and 2N.
**Table S1** Top 20 differentially expressed genes between lowland and upland cultivars linked to genome‐wide association study outlier loci in northern and southern sites.
Please note: Wiley is not responsible for the content or functionality of any Supporting Information supplied by the authors. Any queries (other than missing material) should be directed to the *New Phytologist* Central Office.

## Data Availability

Code used to analyze data in this manuscript can be found on github: https://github.com/avanwallendael/rust_GWAS. Rust sequencing data can be found on the NCBI SRA PRJNA1106013, and switchgrass data under PRJNA622568.
