# Supplementary material for: Local adaptation of both plant and pathogen: an arms‐race compromise in switchgrass rust
Source: New Phytol. 2025 Jun 22;248(3):1527–41. doi: 10.1111/nph.70313 (PMC12489299; doi:10.1111/nph.70313)
Supplement: Supplementary file 1 — Fig. S1 Linkage decay in each switchgrass subpopulation. Fig. S2 Principal component analysis and genome‐wide F ST calculations for rust samples. Fig. S3 Quantile‐quantile plots for northern and southern sites genome‐wide association study. Fig. S4 Top overrepresented gene functions linked to outlier loci in the North and South regions. Fig. S5 Outlier‐linked regions on Chromosomes 3N and 2N. Table S1 Top 20 differentially expressed genes between lowland and upland cultivars linked to genome‐wide association study outlier loci in northern and southern sites. Please note: Wiley is not responsible for the content or functionality of any Supporting Information supplied by the authors. Any queries (other than missing material) should be directed to the New Phytologist Central Office. [file NPH-248-1527-s001.docx]

New Phytologist Supporting Information

Article title:Local adaptation of both plant and pathogen: an arms-race compromise in switchgrass rust

Authors: Acer VanWallendael, Chathurika Wijewardana, Jason Bonnette, Lisa Vormwald, Felix B. Fritschi, Arvid Boe, Shelly Chambers, Robert B. Mitchell, Francis M. Rouquette Jr, Yanqi Wu, Philip A. Fay, Julie D. Jastrow, John T. Lovell, Thomas Juenger, David B. Lowry

Article acceptance date: 22 May 2025

**
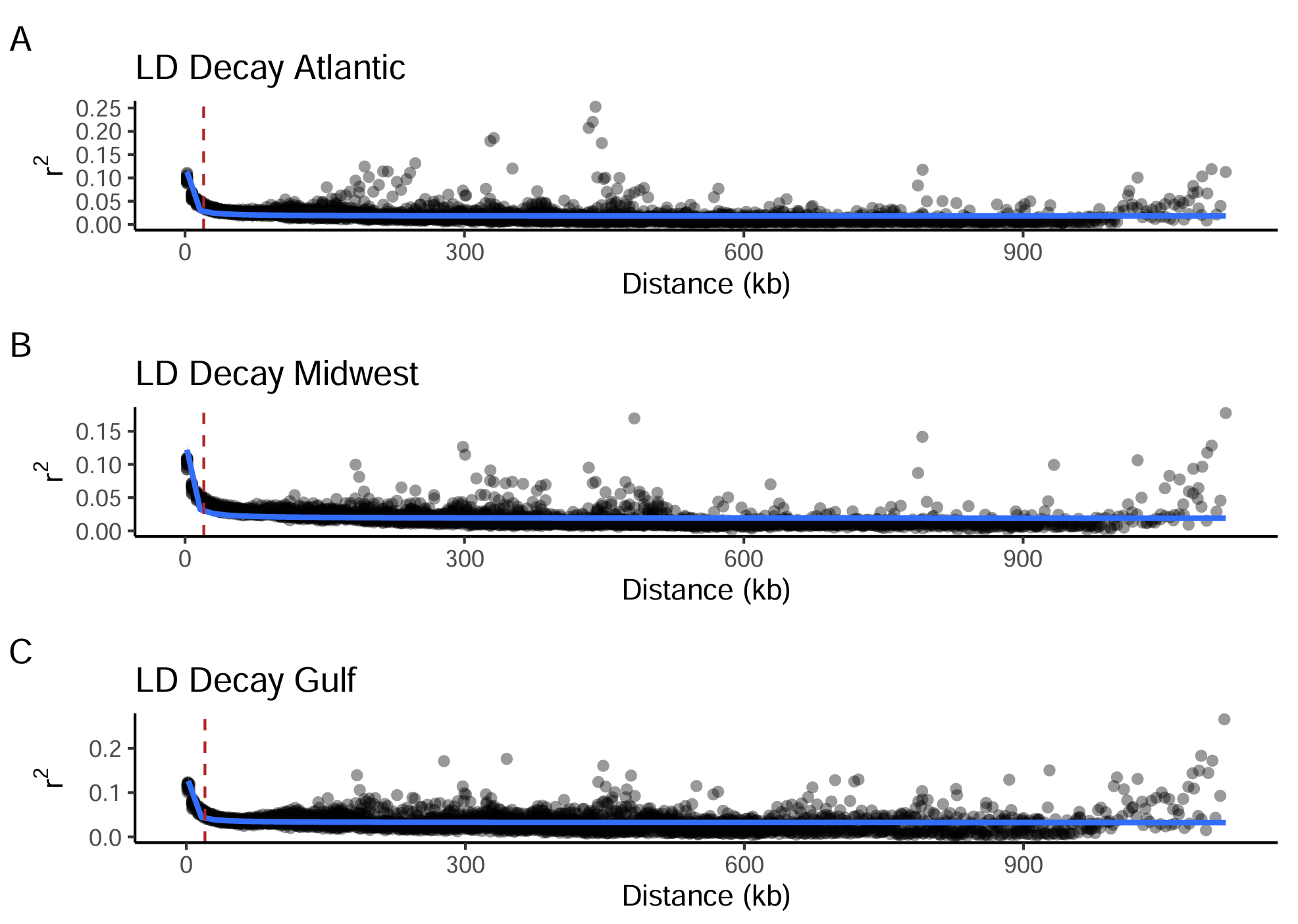
**

**Figure S1:** Linkage decay (LD) in each switchgrass subpopulation. Each point represents the mean r^2^ value for 500 single nucleotide polymorphism bins across distance values. All chromosomes are shown on each of A, B, and C. The blue line indicates a fitted decay curve, and the dashed vertical line shows 20 kilobases, the typical linkage distance used for switchgrass.

A


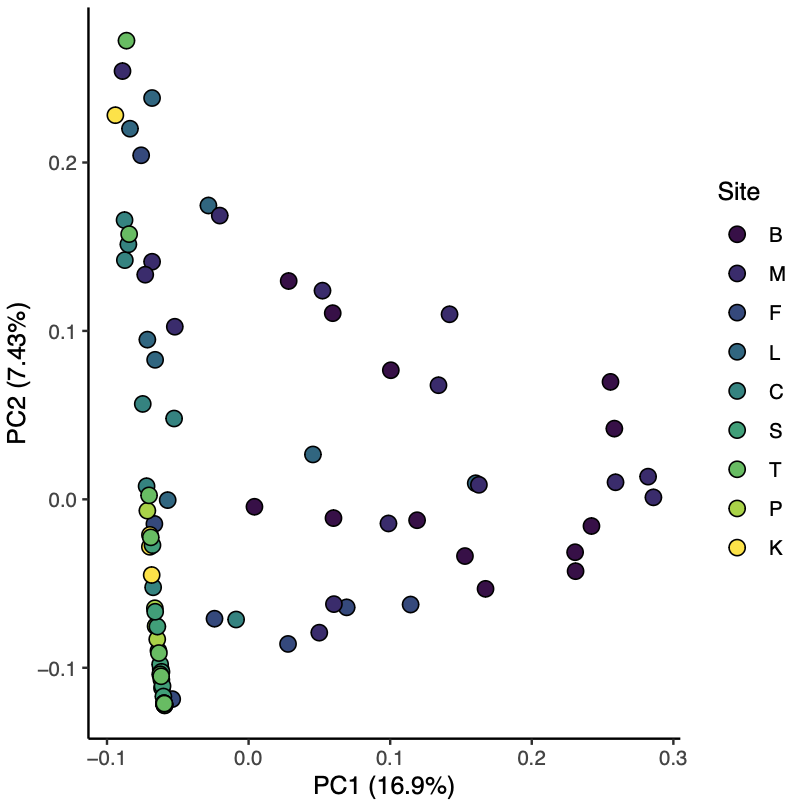


B
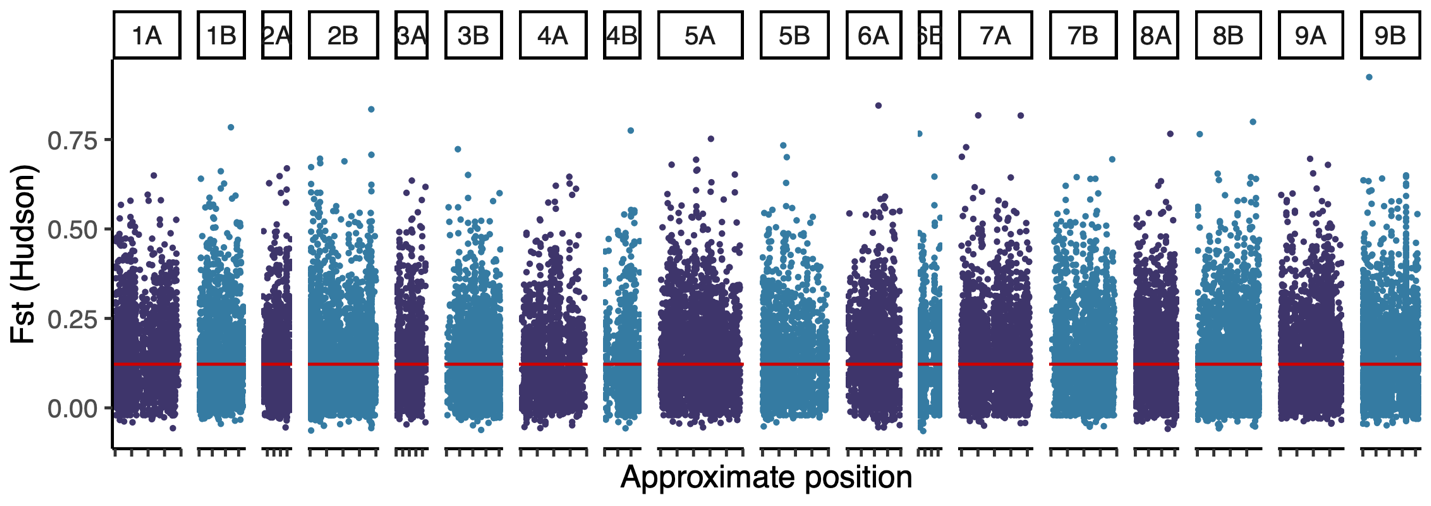


**Figure S2:** Rust genetic diversity **A:** Principal component analysis (PCA) of rust samples, with each point representing the rust collected from a single switchgrass plant. Darker points come from higher latitudes. Axis labels indicate percent variance explained (PVE).Site codes correspond to: B-Brookings, SD; M-Kellogg Biological Station, MI; F-Fermilab, IL; L-Lincoln, NE; C-Columbia, MO; S-Stillwater, OK; T-Temple, TX; P-J.J. Pickle Research Campus, TX; and K-Kingsville, TX. **B**: Sliding-window Hudson’s Fst calculation of the differences between Northern and Southern rust populations. Red horizontal line shows the mean Fst across chromosomes.

**A**
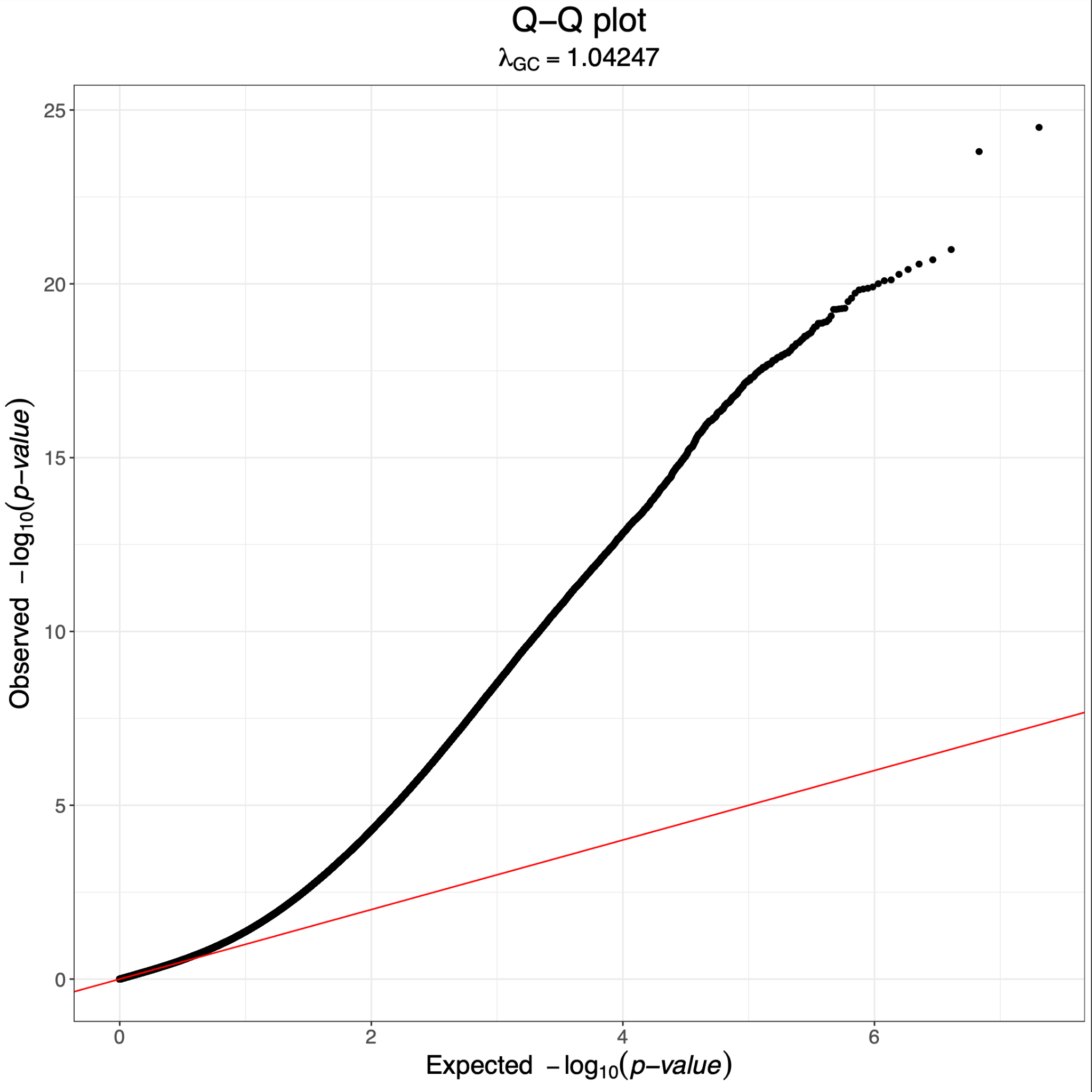


**B**
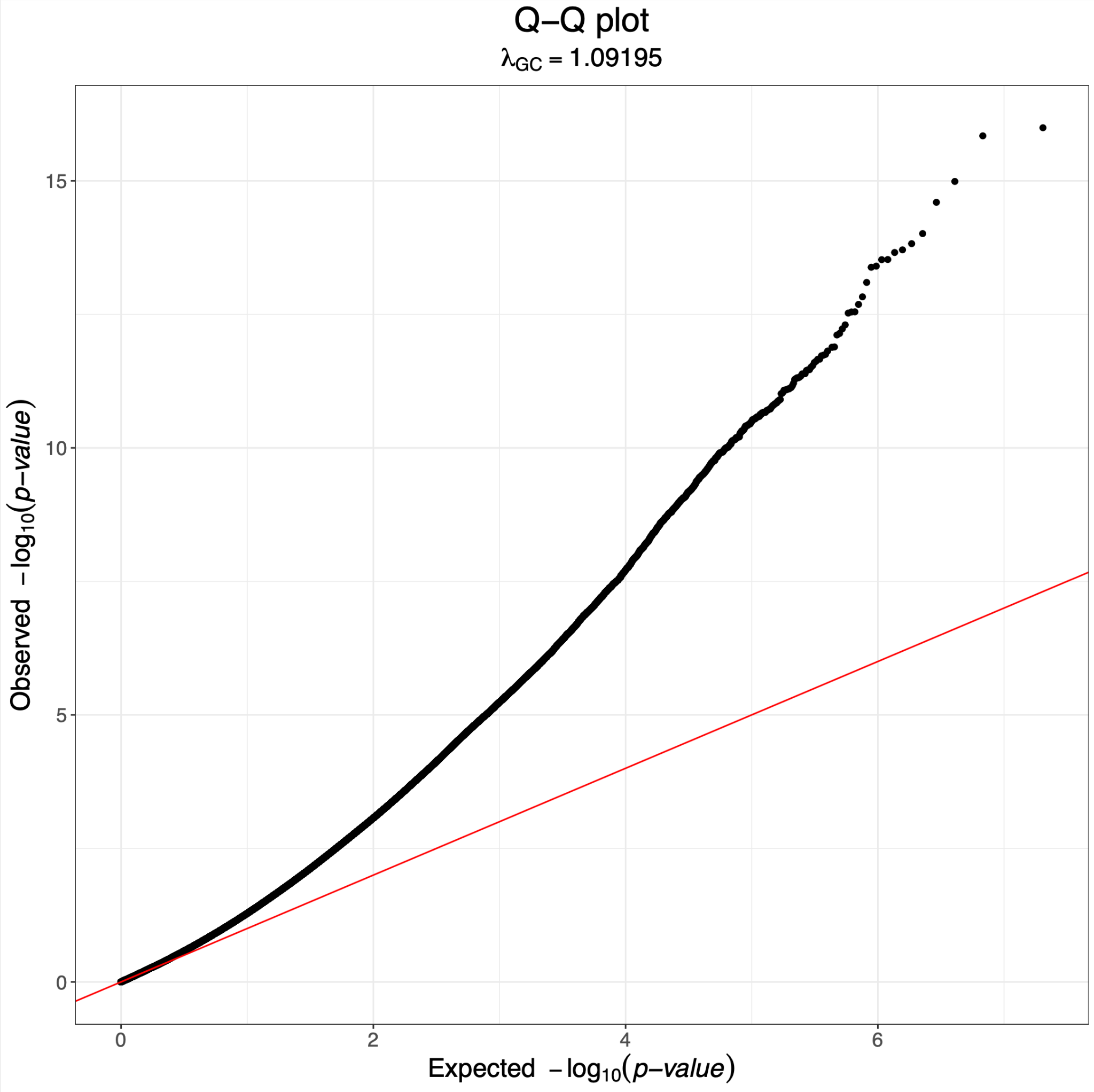


**Figure S3:** Quantile-quantile (Q-Q) plots for northern (A) and southern (B) sites GWAS.

**A.** North overrepresented


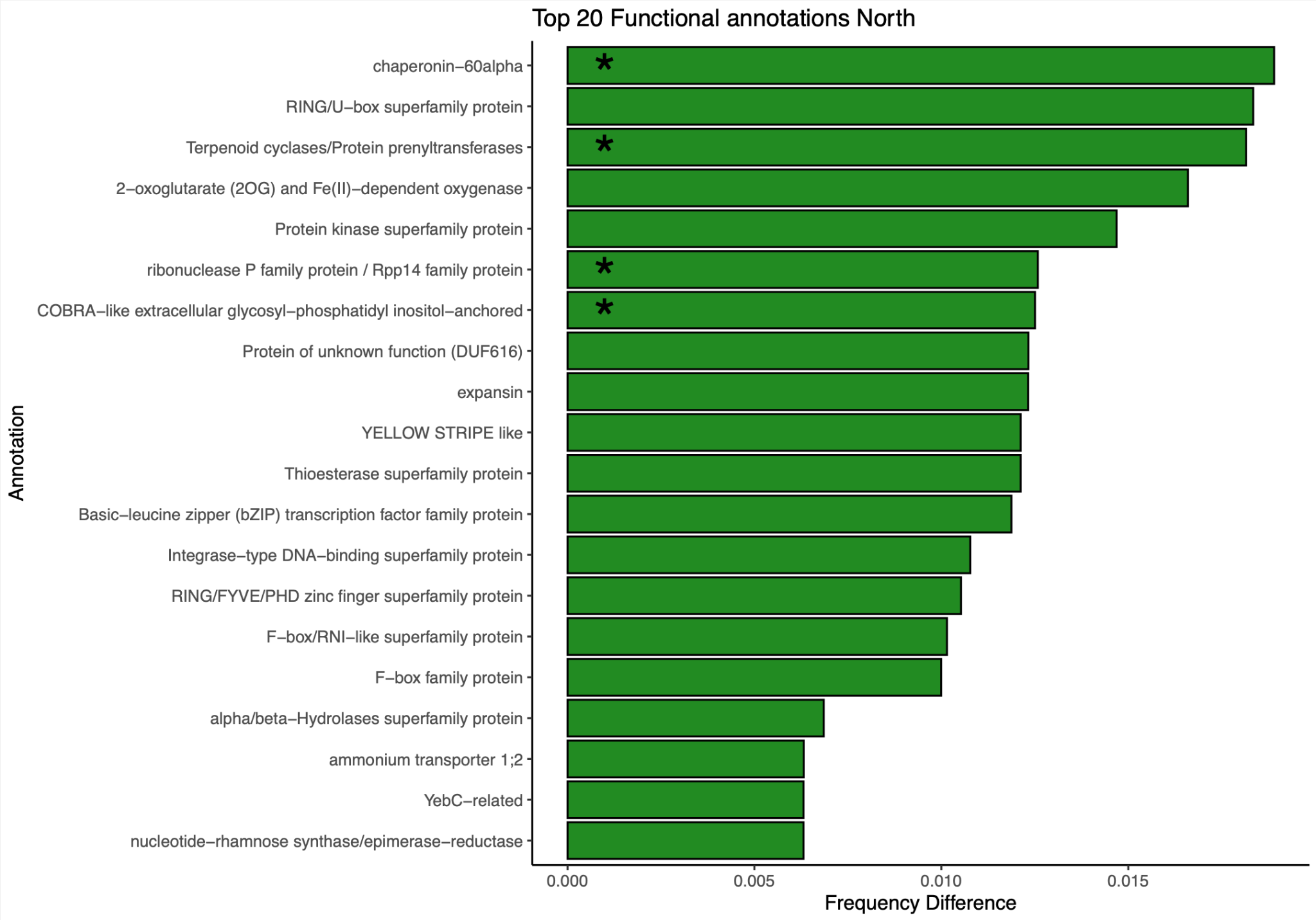


**B.** South overrepresented


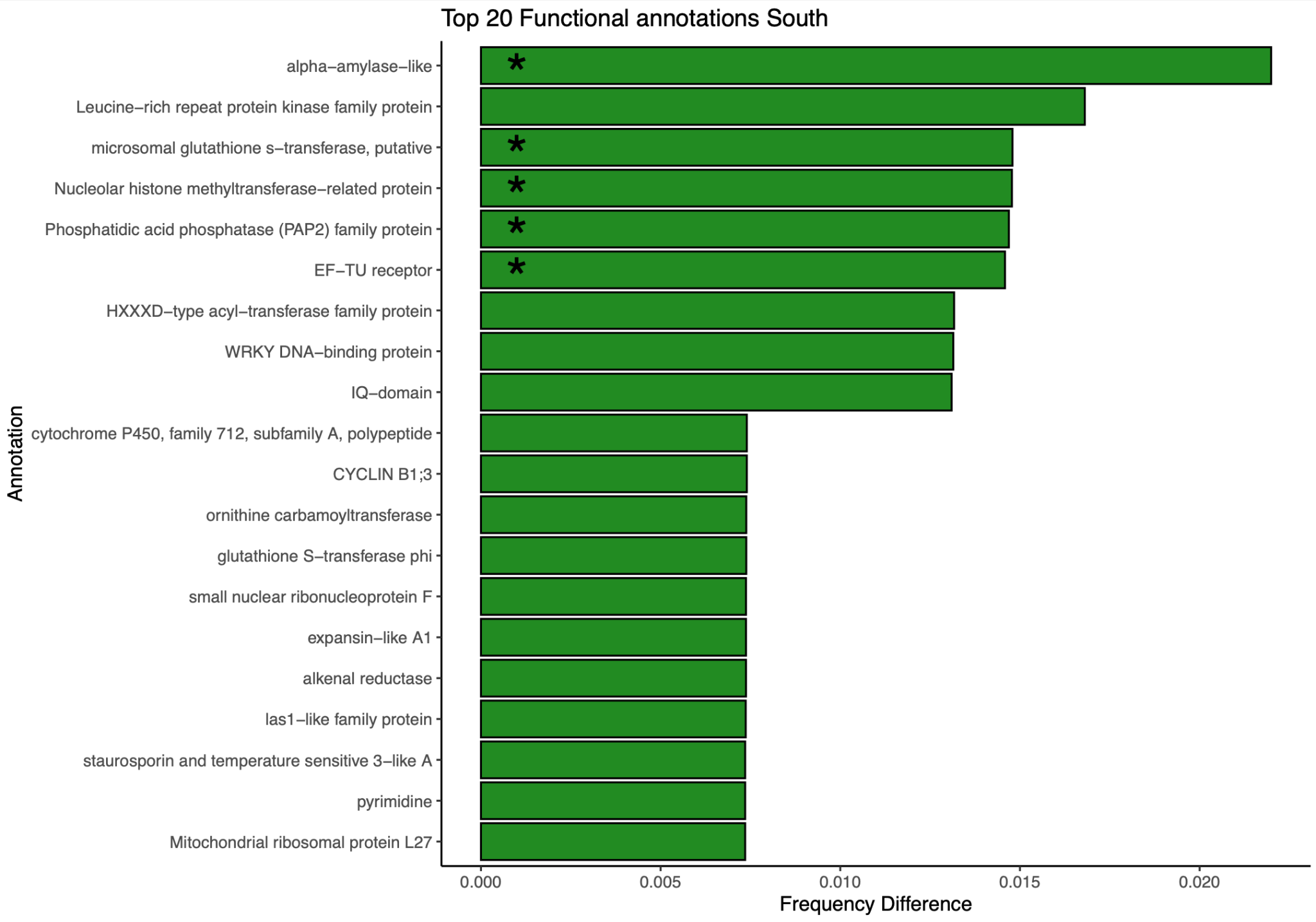


**Figure S4:** Top overrepresented gene functions linked to outlier loci in the North (**A**) and South (**B**) regions. Asterisks indicate annotations that were statistically more commonly linked to top single-nucleotide polymorphisms via bootstrap resampling.


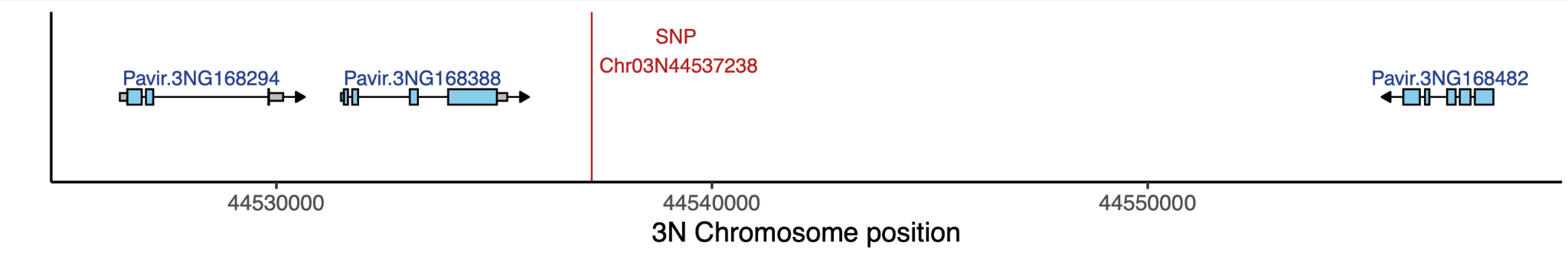

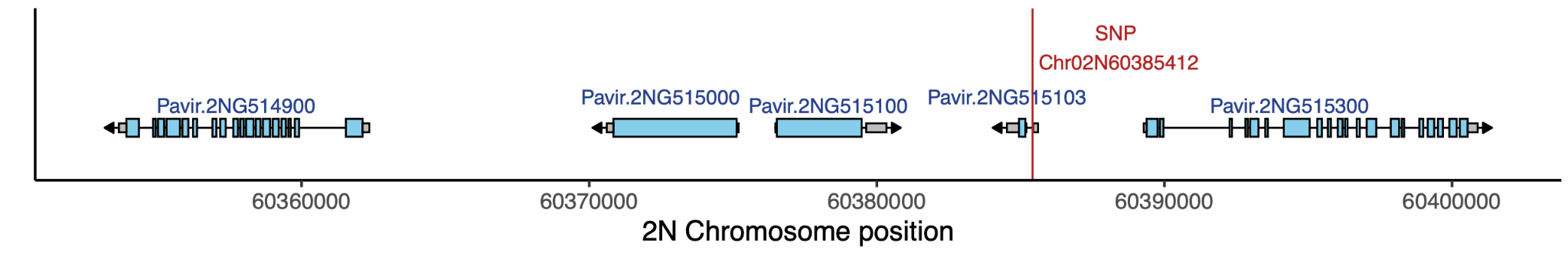


**Figure S5:** Outlier-linked regions on Chromosomes 3N and 2N. Genes Pavir.2NG514900.1, 515000.1, 515100.1, and 515300.1 have functions related to drug- or disease-resistance. Red vertical lines indicate outlier polymorphism positions from genome-wide associations.

# Supplemental tables

Table S1: Top 20 differentially expressed (DE) genes between lowland and upland cultivars linked to genome-wide association study outlier loci in northern and southern sites. The first column indicates the -log_10_ of an adjusted p-value from a Wald test for differential expression between ecotype.

| **Top DE genes North** | |  |  |
| --- | --- | --- | --- |
| **DE.log10p** | **Predicted.Function** | **GeneID** | **GWAS.log10p** |
| 122.526 |  | Pavir.7NG105945 | 17.902 |
| 75.144 | RING/U-box superfamily protein | Pavir.1NG505000 | 17.453 |
| 32.984 | squamosa promoter-binding protein-like 12 | Pavir.5KG215300 | 20.414 |
| 30.654 | Terpenoid cyclases/Protein prenyltransferases superfamily protein | Pavir.1KG382200 | 20.114 |
| 23.737 | hydroxyproline-rich glycoprotein family protein | Pavir.2KG386500 | 18.186 |
| 18.298 |  | Pavir.7NG018100 | 17.951 |
| 14.275 | Phosphoinositide phosphatase family protein | Pavir.1KG521340 | 19.264 |
| 14.275 | Protein kinase superfamily protein | Pavir.3NG140845 | 17.411 |
| 14.110 |  | Pavir.2NG515103 | 23.804 |
| 12.404 |  | Pavir.9NG187100 | 18.076 |
| 12.313 | ammonium transporter 1;2 | Pavir.1NG352400 | 17.227 |
| 10.890 | F-box family protein | Pavir.9KG451700 | 18.191 |
| 9.303 | peroxin 11A | Pavir.9NG683114 | 19.076 |
| 9.092 | Pentatricopeptide repeat (PPR) superfamily protein | Pavir.1KG506800 | 18.431 |
| 8.883 |  | Pavir.1KG549101 | 20.569 |
| 8.872 | Transducin family protein / WD-40 repeat family protein | Pavir.2KG386500 | 18.186 |
| 8.138 | ribonuclease P family protein / Rpp14 family protein | Pavir.7NG018200 | 17.951 |
| 8.089 | Protein kinase superfamily protein | Pavir.9NG186800 | 18.076 |
| 7.676 | Terpenoid cyclases/Protein prenyltransferases superfamily protein | Pavir.1KG382115 | 17.391 |
| 7.594 | 2-oxoglutarate (2OG) and Fe(II)-dependent oxygenase superfamily protein | Pavir.1NG460500 | 19.911 |

| **Top DE genes South** | |  | |  |  |
| --- | --- | --- | --- | --- | --- |
| **DE.log10p** | **Predicted.Function** | | **GeneID** | **GWAS.log10p** | |
| 24.883 | small nuclear ribonucleoprotein F | | Pavir.5NG167600 | 12.547 | |
| 20.847 | FRIGIDA-like protein | | Pavir.2KG258800 | 11.390 | |
| 18.436 |  | | Pavir.3KG041145 | 10.702 | |
| 18.224 |  | | Pavir.1NG427100 | 13.098 | |
| 17.370 | Galactosyltransferase family protein | | Pavir.4KG405400 | 10.899 | |
| 16.931 | bidirectional amino acid transporter 1 | | Pavir.3NG006300 | 10.656 | |
| 16.833 | Homeodomain-like protein with RING/FYVE/PHD-type zinc finger domain | | Pavir.5NG495200 | 11.104 | |
| 16.544 | phytochrome C | | Pavir.9KG462324 | 10.664 | |
| 12.988 |  | | Pavir.3KG554100 | 10.905 | |
| 12.774 | alpha-amylase-like | | Pavir.3NG274900 | 12.113 | |
| 12.388 | CYCLIN B1;3 | | Pavir.5NG167700 | 12.547 | |
| 10.903 |  | | Pavir.9NG458114 | 10.629 | |
| 10.694 | RNI-like superfamily protein | | Pavir.4KG405200 | 10.899 | |
| 10.640 | alpha-amylase-like | | Pavir.3NG274800 | 12.113 | |
| 10.333 | wall associated kinase 3 | | Pavir.9KG404732 | 11.465 | |
| 10.136 | dicer-like 3 | | Pavir.9NG292600 | 10.587 | |
| 10.078 |  | | Pavir.6KG013176 | 10.799 | |
| 9.978 | WRKY DNA-binding protein 40 | | Pavir.2KG335400 | 13.659 | |
| 9.155 | Malectin/receptor-like protein kinase family protein | | Pavir.9KG404600 | 11.465 | |
| 9.125 | pyrimidine 2 | | Pavir.5NG543800 | 10.773 | |
